# Trends and Outcomes of Metabolic Surgery in Adolescents with BMI ≥ 50 vs < 50 kg/m^2^: A Retrospective Study Using the MBSAQIP Database

**DOI:** 10.1007/s11695-025-08080-4

**Published:** 2025-07-19

**Authors:** Pattharasai Kachornvitaya, Mélissa V Wills, Juan S Barajas-Gamboa, Salvador Navarrete, Ricard Corcelles, Andrew Strong, Suthep Udomsawaengsup, Matthew Kroh, Jerry Dang, Valentin Mocanu

**Affiliations:** 1 Digestive Disease Institute, Cleveland Clinic, Cleveland, Ohio, United States; 2 Cleveland Clinic Lerner College of Medicine, Cleveland, United States; 3 Digestive Diseases Institute, Cleveland Clinic Abu Dhabi, Abu Dhabi, United Arab Emirates; 4 Department of Surgery, Faculty of Medicine, Chulalongkorn University, Bangkok, Thailand; 5 Treatment of Obesity and Metabolic Disease Research Unit, Faculty of Medicine, Chulalongkorn University, Bangkok, Thailand

**Keywords:** Adolescent obesity, Sleeve gastrectomy, Roux-en-Y gastric bypass, BMI ≥ 50 kg/m^2^, Serious complications, MBSAQIP database

## Abstract

**Background:**

Metabolic and bariatric surgery (MBS) is an effective and increasingly utilized treatment for adolescents with severe obesity, particularly those with BMI ≥ 50 kg/m^2^. However, data on safety and outcomes in this high-risk group remains limited.

**Methods:**

We conducted a retrospective analysis of the MBSAQIP database from 2020–2023, identifying adolescents aged 13–18 years who underwent primary sleeve gastrectomy (SG) or Roux-en-Y gastric bypass (RYGB). Patients were stratified by BMI into < 50 kg/m^2^ and ≥ 50 kg/m^2^ cohorts. Thirty-day outcomes were compared, and multivariable logistic regression was used to identify predictors of serious complications.

**Results:**

Among 3,056 adolescents (0.4% of 692,615 total cases), 2,134 (69.8%) had BMI < 50 and 922 (30.2%) had BMI ≥ 50 kg/m^2^. MBS volume increased by 51% from 2020 to 2023. The BMI ≥ 50 cohort had more comorbidities: diabetes (20.9% vs 14.5%), hypertension (11.8% vs 5.2%), and sleep apnea (32.9% vs 17.9%) (all *p* < 0.001), and longer operative times (82.8 vs 75.5 min, *p* < 0.001). However, serious complication rates were comparable (1.3% vs 1.5%, *p* = 0.668), and no 30-day mortality was reported. RYGB (OR 2.7, 95%CI 1.21–5.94, *p* = 0.015) and non-insulin dependent type 2 diabetes (OR 2.3, 95% CI 1.2–4.58, *p *= 0.016) were the independent predictors of serious complications.

**Conclusions:**

MBS is safe in adolescents, including those with BMI ≥ 50 kg/m^2^, with no increased short-term risk of serious complications. These findings support broader surgical consideration in this high-risk group.

## Introduction

The global rise in adolescent obesity has emerged as a critical public health concern, with severe obesity increasingly diagnosed during early adolescence. The proportion of teenagers with obesity in the United States is 20.6% [1]. Characterized by early onset of obesity associated medical problems such as type 2 diabetes, hypertension, and obstructive sleep apnea, adolescent obesity often persists into adulthood, contributing to long-term cardiovascular and metabolic disease burden [2]. Metabolic and bariatric surgery (MBS) has gained traction as an effective and safe intervention for adolescents with severe obesity, especially when non-surgical therapies fail [3]. Clinical outcomes from the Teen-Longitudinal Assessment of Bariatric Surgery (Teen-LABS) study and other registries demonstrate that MBS in adolescents leads to significant and sustained weight loss, as well as resolution of associated medical problems [4, 5].

Despite growing evidence and endorsement from professional societies, including the American Society for Metabolic and Bariatric Surgery (ASMBS) and the American Academy of Pediatrics (AAP), the use of MBS in adolescents remains disproportionately low [6, 7]. This is particularly true for adolescents with BMI ≥ 50 kg/m^2^, a group at especially high risk for adverse health outcomes and reduced responsiveness to lifestyle interventions. A recent study revealed that 0.18% of children and adolescents (aged 10–19 years) with obesity get MBS annually and youth cases (aged 15–24 years) accounted for only 3.7% of all bariatric procedures in the MBSAQIP registry between 2015 and 2018, despite representing a growing segment of the severely obese population [8, 9].

While prior studies have demonstrated the perioperative safety of MBS in adolescents [10, 11], none have specifically focused on those with BMI ≥ 50 kg/m^2^. Concerns remain regarding the safety and complexity of performing MBS in this high-risk subgroup, which often presents significant technical challenges and prolonged operative times. Although BMI ≥ 50 kg/m^2^ has been associated with increased perioperative risk in adults, the extent to which this applies to adolescents remains unclear and underexplored. These patients typically have a higher burden of associated medical problems, including type 2 diabetes, hypertension, dyslipidemia, and obstructive sleep apnea. Furthermore, a BMI ≥ 50 kg/m^2^ is an independent risk factor for serious complications and increased 30-day postoperative mortality [12]. Previous analyses of national datasets have provided important insights into postoperative outcomes. Nevertheless, they have largely not stratified findings by BMI category or evaluated the effect of extreme obesity on complication rates.

The present study aims to address these gaps by evaluating the short-term safety and utilization trends of MBS among adolescents with BMI ≥ 50 kg/m^2^ compared to those with lower BMI. Using a large, nationally representative cohort from the MBSAQIP registry (2020–2023), we assess 30-day postoperative outcomes and identify predictors of serious complications. Our findings seek to inform clinical decision-making, potentially expand access to MBS, and support guideline-directed care for adolescents with morbid obesity who face the highest risk for premature obesity associated medical problems and mortality.

## Method

### Data Source

An explorative retrospective analysis of the MBSAQIP data registry was performed from 2020 to 2023. The MBSAQIP currently captures clinical data from nearly 1,000 accredited American and Canadian centers. The data registry prospectively collects data and contains standardized pre-, intra-, and post-operative variables specific to bariatric surgery patients. The MBSAQIP centers have rigorous semiannual data audits to ensure data integrity, with centers higher than a 5% data disagreement rate not included in the final registry and subject to further educational training and audits. This study used de-identified data and was exempt from Institutional Review Board approval according to institutional policies.

### Patient Variables and Population

All adolescents aged 13–18 years who underwent primary sleeve gastrectomy (SG) or Roux-en-y gastric bypass (RYGB) between 2020–2023 were included in this retrospective cohort study. Individuals'BMI was stratified into two cohorts: BMI < 50 kg/m^2^ and BMI ≥ 50 kg/m^2^. 30-day outcomes were evaluated between cohorts. Patients who underwent prior bariatric surgery, emergency surgery, conversion procedures, or revision procedures were excluded.

Patients who underwent primary SG and RYGB were identified using Current Procedural Terminology (CPT) codes 43,775 and 43,644, respectively.

Basic demographic data including types of surgery (SG or RYGB), age, sex, race, body mass index (BMI), functional status, American Society of anesthesiologists (ASA) Physical Status classification and smoking status were collected. Patient associated medical problems included the following: type 2 diabetes, hypertension, gastroesophageal reflux disease (GERD), hyperlipidemia, obstructive sleep apnea, and renal insufficiency. Patient history included previous venous thromboembolism (VTE), dialysis, immunosuppressive therapy, previous major cardiac surgery and preoperative therapeutic anticoagulant. Perioperative data including operative length and drain placement.

### Objectives and Composite Outcome Definitions

The primary objective of this study was to determine the rate of serious complications and mortality of SG and RYGB in adolescents with BMI ≥ 50 kg/m^2^ compared to those with BMI < 50 kg/m^2^. Secondary objectives included characterizing the prevalence and trends in delivery of bariatric care in these cohorts as well as identifying independent predictors of serious complications.

Patients with at least one of the following complications within 30 days of surgery were defined as having a serious complication: anastomotic leak, postoperative bleeding, reoperation, non-operative intervention, cardiac event (cardiac arrest, myocardial infarction, or cardiopulmonary resuscitation), pneumonia, unplanned intubation, acute kidney injury, venous thromboembolism, deep surgical site infection or wound disruption, sepsis, cerebrovascular accident.

### Statistical Analysis

Categorical variables were summarized as percentages, while continuous variables were reported as weighted means ± standard deviations (SD). Baseline differences between cohorts were assessed using chi-squared tests for categorical variables and independent sample t-tests for continuous variables. To compare outcomes between adolescents with BMI ≥ 50 kg/m^2^ and BMI < 50 kg/m^2^ cohorts, univariate logistic regression analyses were conducted. Multivariable logistic regression was then used to identify independent predictors of serious complications. Variables with a *p*-value < 0.05 in univariate analysis were included in the multivariable model. Patient characteristics and operative time were also incorporated. Collinearity among variables was assessed using variance inflation factors (VIFs). Model performance was evaluated using the area under the receiver operating characteristic (AUROC) curve for discrimination, and the Brier score for calibration. Data were analyzed using Stata version 17 (StataCorp, College Station, TX, USA).

## Results

### Patient Baseline Characteristics

A total of 3,056 adolescents (0.44% of 692,615 total cases) underwent bariatric surgery between 2020 and 2023, of which 2,134 (69.9%) had a BMI < 50 kg/m^2^ and 922 (30.1%) had a BMI ≥ 50 kg/m^2^ (Table 1). A significantly lower proportion of females was observed in the BMI ≥ 50 kg/m^2^ cohort (63.8%) compared to the BMI < 50 kg/m^2^ cohort (72.5%;* p* < 0.001) with racial distribution also differing significantly, with a higher proportion of Black adolescents in the BMI ≥ 50 kg/m^2^ cohort (31.6% vs. 17.5%, *p* < 0.0001). The prevalence of obesity associated medical problems was generally higher in adolescents with BMI ≥ 50 kg/m^2^ with higher rates of diabetes (20.9% vs. 14.5%; *p* < 0.001), hypertension (11.8% vs. 5.2%; *p *< 0.001), and obstructive sleep apnea (32.9% vs. 17.9%; *p* < 0.001).

**Table 1:** Baseline characteristics of the adolescents undergoing bariatric surgery

	Adolescents with BMI < 50 kg/m^2^ (*n* = 2,134)	Adolescents with BMI ≥ 50 kg/m^2^ (*n* = 922)	*p*-value
Bariatric procedures			0.285
RYGB	1009 (47.3)	417 (45.2)	
SG	1,125 (52.7)	506 (54.8)	
Age (years, mean ± SD)	17.0 ± 1.2	17.0 ± 1.2	0.751
Female	1548 (72.5)	589 (63.8)	< 0.001
Race			< 0.001
White	1,119 (52.4)	402 (49.7)	
Black or African American	373 (17.5)	292 (31.6)	
Other	642 (30.1)	229 (24.8)	
Body mass index (kg/m^2^, mean ± SD)	43.1 ± 4.1	56.9 ± 6.3	< 0.001
Functional Status			0.789
Independent	2,127 (99.7)	917 (99.7)	
Partially Dependent	6 (0.3)	3 (0.3)	
Dependent	1 (0.05)	0 (0)	
Smoker	43 (2)	13 (1.4)	0.251
Diabetes			< 0.001
None or Diet Controlled	1,824 (85.5)	730 (79.1)	
Non-insulin Dependent	275 (12.9)	162 (17.5)	
Insulin Dependent	35 (1.6)	31 (3.4)	
Hypertension	110 (5.2)	109 (11.8)	< 0.001
Gastroesophageal reflux disease	185 (8.7)	90 (9.8)	0.337
Dyslipidemia	75 (3.5)	30 (3.3)	0.713
Obstructive sleep apnea	383 (17.9)	304 (32.9)	< 0.001
Immunosuppressive therapy	19 (0.9)	7 (0.8)	0.715
Renal Insufficiency	4 (0.2)	0 (0)	0.188
History of venous thromboembolism	4 (0.2)	3 (0.2)	0.465
Therapeutic anticoagulation use	6 (0.3)	3 (0.3)	0.837
Previous major cardiac surgery	6 (4.8)	7 (1.9)	0.079

*RYGB* Roux-en-Y gastric bypass, *SG* Sleeve gastrectomy, *ASA* American Society of Anesthesiologists

### Trends and Outcomes Associated with Adolescent Bariatric Surgery

**Fig. 1:**
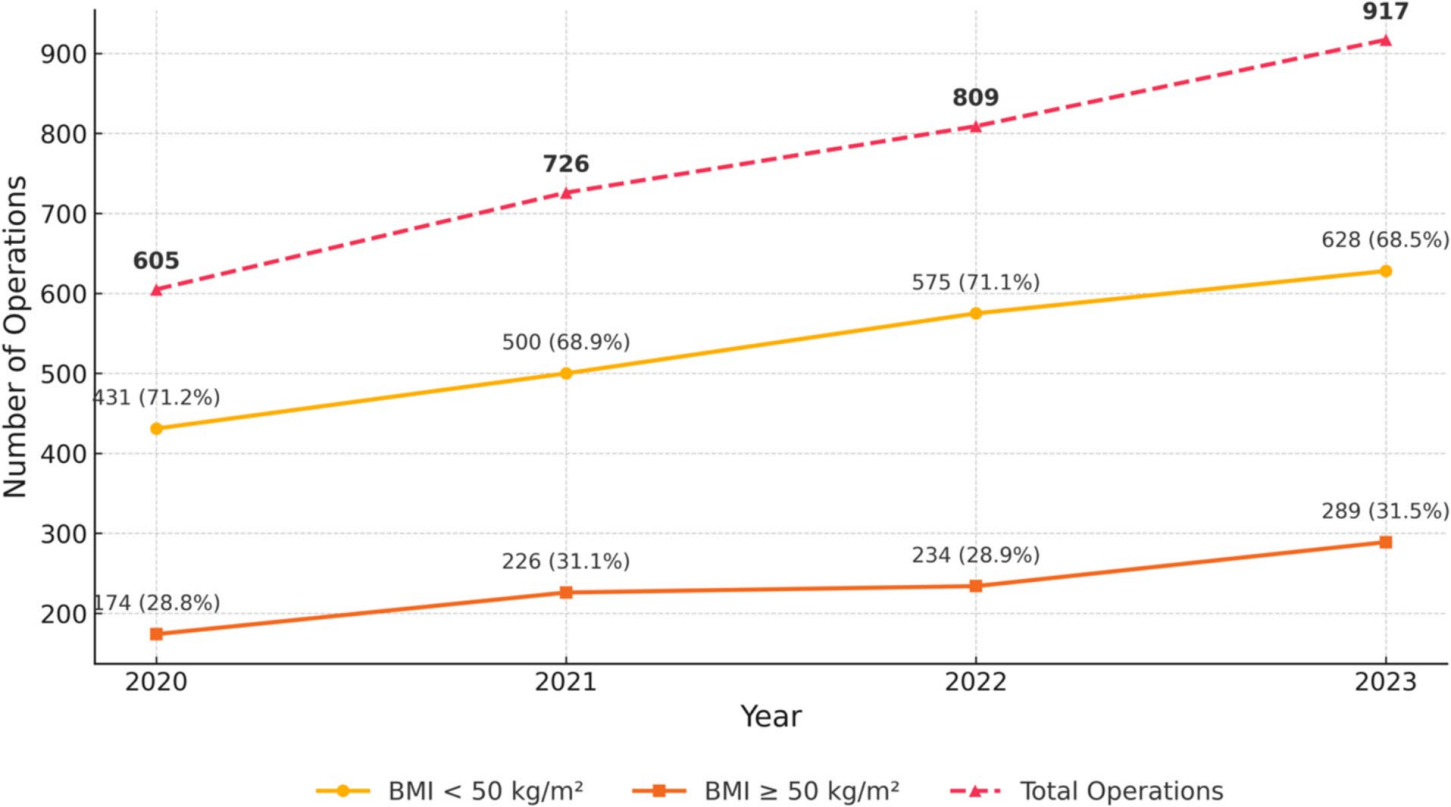
Trends in bariatric surgery among adolescents by BMI group between 2020 and 2023

Figure 1 outlines the trend in adolescent bariatric surgery during the 2020–2023 period. The number of surgeries increased over time in both cohorts, with no significant difference in the annual distribution between the BMI cohorts (*p* = 0.5). In 2023, the proportion of surgeries peaked at 29.4% and 31.3% in the BMI < 50 and ≥ 50 kg/m^2^ cohorts, respectively.

Adolescents with BMI ≥ 50 kg/m^2^ had a longer mean operative time (82.8 ± 49.9 vs. 75.5 ± 37.7 min; *p *< 0.001). Postoperative complication rates, including anastomotic leak, postoperative bleeding, and bowel obstruction, were similar between the cohorts. There were no significant differences in the rates of 30-day reoperation, non-operative interventions, or hospital readmissions. The overall 30-day serious complication rate was 1.44% across the entire cohort. No 30-day mortality was observed in either group (Table 2).

**Table 2:** Perioperative details and 30-day postoperative complications

	Adolescents with BMI < 50 kg/m^2^ (*n* = 2,134)	Adolescents with BMI ≥ 50 kg/m^2^ (*n* = 922)	*p*-value
Operative details			
Operative Length (mins)	75.5 ± 37.7	82.8 ± 49.9	< 0.001
Drain placement	56 (2.6)	33 (3.6)	0.151
Surgical complications, n (%)			
Leaks	2 (0.1)	1 (0.1)	0.906
Postoperative bleeding	7 (0.3)	2 (0.2)	0.602
Bowel obstruction	1 (0.05)	0 (0)	0.511
Reoperation	10 (0.5)	3 (0.3)	0.575
Non-operative intervention	17 (0.8)	5 (0.5)	0.444
Readmission	57 (2.7)	17 (1.8)	0.171
Cardiac events	1 (0.05)	0 (0)	0.511
Infectious complications, n (%)			
Pneumonia	2 (0.1)	1 (0.1)	0.906
Deep SSI	0 (0)	0 (0)	-
Wound Disruption	3 (0.1)	2 (0.2)	0.633
Sepsis	1 (0.05)	2 (0.2)	0.169
Medical complications, n (%)			
Venous Thromboembolism	0 (0)	0 (0)	-
AKI	0 (0)	1 (0.1)	0.128
Composite outcomes, n (%)			
Serious Complications	32 (1.5)	12 (1.3)	0.668
Mortality	0 (0)	0 (0)	-

*AKI* Acute kidney injury, *SSI* superficial site infection

### Multivariable Logistic Regression for Predictors of Serious Complications

After adjustment with multivariable logistic regression, the higher BMI cohort analyzed as a categorical variable was not an independent risk factor (OR 0.74, 95% CI 0.37–1.47, *p* = 0.386) for serious complications following bariatric surgery. Significant independent risk factors associated with serious complication were RYGB (OR 2.7, 95%CI 1.21–5.94, *p* = 0.015) and non-insulin dependent diabetes (OR 2.3, 95% CI 1.2–4.58, *p* = 0.016). Other variables, including female sex, and race were not significantly associated with increased risk. The model had an AUROC of 0.67 and Brier score 0.01. (Table 3).

**Table 3:** Predictors of serious complications in adolescents undergoing bariatric surgery

	Adjusted odds ratio^*^	95% Confidence Interval	*p*-value
Higher BMI (per 5 kg/m^2^)	0.74	0.37—1.47	0.386
Female	1.3	0.63—2.66	0.478
Race			
Black	1.7	0.84—3.44	0.143
Other	0.99	0.46—2.15	0.926
RYGB vs SG	2.7	1.21—5.94	0.015
Diabetes			
Non-insulin dependent vs. non-diabetic/diet controlled	2.31	1.17—4.58	0.016
Insulin dependent vs. non- diabetic/diet controlled	2.2	0.5—9.67	0.297

*BMI* body mass index, *RYGB* Roux-en-Y gastric bypass, *SG* Sleeve gastrectomy

*Adjusted for age, hyperlipidemia, obstructive sleep apnea, previous myocardial infarction, and dialysis dependency

## Discussion

This study represents the largest known analysis of adolescent bariatric surgery using data from a major North American registry between 2020 and 2023, derived from the MBSAQIP database. Despite a higher burden of comorbidities—including significantly increased rates of type 2 diabetes, hypertension, and obstructive sleep apnea—adolescents with BMI ≥ 50 kg/m^2^ experienced 30-day complication rates comparable to those with lower BMI. These findings underscore the role of MBS as a safe and effective intervention for adolescents with severe obesity, including those at the highest levels of BMI. Nevertheless, we emphasize that surgical candidacy should not be determined by BMI alone; rather, it should reflect a multidisciplinary evaluation, as mandated by accredited centers.

Our results are consistent with prior evidence demonstrating the safety of MBS in adolescents. Large studies such as Teen-LABS and analyses of the MBSAQIP registry have previously reported low perioperative morbidity and mortality rates in adolescent populations undergoing laparoscopic sleeve gastrectomy (LSG) or laparoscopic Roux-en-Y gastric bypass (LRYGB) [5, 11, 13]. Previous cohorts such as Teen-LABS included limited numbers of adolescents with BMI ≥ 50 kg/m^2^, restricting the ability to specifically evaluate this high-risk subgroup. Notably, our findings addressing this gap by extending this safety profile through a larger sample that allows for stratified analysis in adolescents with BMI ≥ 50 kg/m^2^, a subgroup historically underrepresented in prior studies. The comparable complication rates, despite longer operative times and higher preoperative risk profiles, suggest that BMI alone should not in of itself be a contraindication for surgery in this age group [10].

The predominance of LSG over RYGB in our study mirrors national trends both for adults and children, and is likely attributable to its shorter operative duration, technical simplicity, and favorable safety profile [11, 13, 14], as well as concerns about the long-term risks of malabsorptive procedures like RYGB—particularly in adolescents, who face prolonged cumulative exposure. Previous investigations have shown that LSG is associated with lower rates of early complications compared to RYGB, particularly in younger patients. Although RYGB was associated with serious complications in the adjusted model (OR 2.7, 95%CI 1.21–5.94, *p* = 0.015), we emphasize that RYGB remains an essential surgical option and should be considered for adolescents, particularly those with severe gastroesophageal reflux disease or poorly controlled type 2 diabetes. Further long-term nutritional and metabolic data, however, is needed to better delineate the ideal operation for this population given the increased risk of malnutrition following RYGB which our work was not able to capture or evaluate.

Our findings highlight that type 2 diabetes was the only independent factor associated with serious complications. Both RYGB and SG achieve remission of insulin resistance and type 2 diabetes in at least 90% of adolescents and should be considered primary therapeutic options for children with type 2 diabetes and severe obesity. Childhood-onset type 2 diabetes and insulin resistance should be regarded as strong indications for MBS [15, 16]. These findings underscore the importance of involving endocrinologists in perioperative management to optimize outcomes and suggest that adolescents with type 2 diabetes may benefit from more intensive preoperative optimization and postoperative monitoring regardless of BMI category.

Over the four-year period, we observed a 51% increase in MBS procedures among adolescents, with approximately one-third of the cohort presenting with a BMI ≥ 50 kg/m^2^. However, this increase should be interpreted in the context of reduced surgical activity during the early COVID-19 pandemic and the subsequent post-pandemic rebound, particularly for elective procedures. Despite the increasing utilization of MBS, adolescents remain underrepresented among surgical candidates relative to the prevalence of severe obesity in this age group [17]. In our cohort, Black adolescents were disproportionately represented among those with BMI ≥ 50 kg/m^2^ and may reflect the greater burden of extreme obesity among racial minorities. Although race was not an independent predictor of serious complications, the overrepresentation of Black adolescents in the BMI ≥ 50 kg/m ^2^ highlights significant racial disparities in extreme obesity. This finding, coupled with previous studies showing higher readmission rates in patients [18], underscores the need to address systemic barriers to surgical access, including limited referral patterns, restrictive insurance policies, and implicit bias. Efforts should be directed toward advocating for equitable access to MBS across all racial and ethnic groups.

Evidence increasingly supports the long-term benefits of adolescent MBS. Studies such as FABS-5 + have demonstrated durable weight loss and remission of associated medical problems up to 12 years postoperatively [4, 19]. Early intervention, as endorsed by the ASMBS and the American Academy of Pediatrics, may improve outcomes and reduce long-term morbidity [6, 7]. Cost-effectiveness analyses further highlight the value of timely MBS, particularly among adolescents with a BMI ≥ 50 kg/m^2^, who are at heightened risk for early-onset cardiovascular disease and metabolic dysfunction [20, 21]. Our findings support the safety and efficacy of MBS as an early intervention strategy in adolescents with obesity, consistent with current ASMBS pediatric metabolic and bariatric surgery guidelines [6]. The particularly high burden of obesity associated medical problems in adolescents with BMI ≥ 50 kg/m^2^ observed in our study further supports the case for early surgical intervention in this high-risk group.

This study has several limitations. The MBSAQIP database is de-identified with data only available at the center specific level for quality assurance programs so we are not able to adjust for hospital volume, surgeon experience or more nuanced technical factors that could confound our findings. It is also restricted to 30-day postoperative outcomes and does not capture long-term data on weight loss, comorbidity resolution, revisional requirements, nutritional deficiencies—particularly in RYGB patients—the impact of SG on GERD incidence, or psychosocial and quality of life outcomes, all of which are particularly relevant for the adolescent population undergoing major surgery during a critical developmental period. Socioeconomic variables, which may influence both access to care and postoperative adherence, are also not captured. Additionally, the database does not include center- or surgeon-specific data, and all data were extracted from North American MBSAQIP centers which are subject to rigorous accreditation protocols, and thus may limit the generalizability of the findings. Although this is the single largest cohort study evaluating outcomes within the adolescent population to date, it is possible that it is underpowered to detect smaller effect sizes or that there are other uncaptured covariates which may confound our findings.

Despite these limitations, our study offers the most comprehensive evaluation to date of the short-term safety of MBS in adolescents with BMI ≥ 50 kg/m^2^ undergoing surgery at accredited MBSAQIP centers. Future prospective long-term studies are required to better characterize the safety and metabolic implications of bariatric surgery in the adolescent population.

## Conclusion

MBS is an increasingly utilized treatment strategy for adolescents with severe obesity, including those with BMI ≥ 50 kg/m and in this high-risk subgroup, short-term 30-day outcomes were comparable to adolescents with lower BMI. However, while these findings align with current clinical guidelines and support broadening access to MBS, they should be interpreted with caution in the context of important limitations, including the lack of long-term outcome data, psychosocial metrics, and center-level variables. Future research should prioritize prospective studies to evaluate long-term efficacy, nutritional and psychosocial outcomes, and health equity impacts to guide patient-centered decision-making and optimize care delivery in this population.

## Data Availability

No datasets were generated or analysed during the current study.
